# Supplementary material for: Drivers of the Ectoparasite Community and Co-Infection Patterns in Rural and Urban Burrowing Owls
Source: Biology (Basel). 2022 Jul 29;11(8):1141. doi: 10.3390/biology11081141 (PMC9405203; doi:10.3390/biology11081141)
Supplement: Supplementary file 1 [file biology-11-01141-s001.zip › biology-1816623-supplementary.pdf]

Checklist of parasites reported from *Athene cunicularia*

**Group and parasite species**	**Gegraphic location**	**References**
**Viruses**		
West Nile virus	California (USA)	Dusek et al. (2010)
**Protists**		
Eimeria sp.	Brazil	da Silva et al. (2009)
Eimeria speotytoi	Brazil	Alfaleh et al. (2012)
Entamoeba sp.	California (USA)	Franson (2017)
Histomonas sp.	Brazil	Andery et al. (2013)
	California (USA)	Franson (2017)
Tetratrichomonas sp.	California (USA)	Franson (2017)
Toxoplasma gondii	south Brazil	Vitaliano et al. (2014)
Trichomonas sp.	California (USA)	Franson (2017)
**Acanthocephala**		
Centrorhynchus sp.	Buenos Aires	Drago et al. (2015)
**Trematoda**		
Echinoparyphium speotyto	Oklahoma (USA)	Buscher (1978)
Maritrema patulus	Canada	Hernandez-Orts et al. (2015)
Maritrema sp.	Florida (USA)	Kinsella et al. (2001)
Microphallus sp.	Florida (USA)	Kinsella et al. (2001)
Neodiplostomun americanum	Florida (USA)	Kinsella et al. (2001)
Neodiplostomun travassosi	Argentina	Drago et al. (2015)
Prosthogonimus ovatus	Florida (USA)	Kinsella et al. (2001)
**Nematoda**		
Cyathostoma sp.	Canada	Redig et al. (1993)
Pelecitus sp.	Brazil	Silva et al. (2014)
Subulura alfensis		
Subulura forcipata	Florida (USA)	Kinsella et al. (2001)
Subulura reclinata	Florida (USA)	Kinsella et al. (2001)
Tetrameres sp.	Brazil	Andery et al. (2013)
Acari		
Argas dalei	Peru	Need et al. (1991)
Dermonoton sp.	USA	Kurey (1976)
	Brazil	de Almeida & Hernandes (2016)
Euschoengastoides gurneyu	Kansas (USA)	Loomis (1956)
Glaucalges sp.	Brazil	de Almeida & Hernandes (2016)
Hypodectes propus	Texas (USA)	Pence & Bergan (1996)
	Brazil	Goulart (2011)
Hyponeocula montanensis	Kansas (USA)	Loomis (1956)
Kramerella major		Philips (2000)
Neobubophilus cunicularius	Paraguay	Skoracki et al. (2016)
Neoschoengastia americana	Kansas (USA)	Loomis (1956)
Ornithodoros parkeri	Washington (USA)	Jellison (1940)
Proctophyllodes polyxenus	Nebraska (USA)	Atyeo & Braasch (1966)
Rhinoecius bisetosus	Brazil	Mascarenhas et al. (2018)
Sternostoma augei	Brazil	Mascarenhas et al. (2018)
Tytodectes speotyto	Texas (USA)	Pence & Bergan (1996)
**Phthiraptera**		
Colpocephalum pectinatum	Nebraska (USA)	Thompson (1950)
	Argentina	Daciuk et al. (1981)
	Texas (USA)	Skoruppa et al. (2006)
	Brazil	Oliveira da Silva et al. (2009)
	Mexico	Bolaños-García et al. (2018)
Strigiphilus speotyti	Nebraska, Colorado (USA)	Thompson (1950)
	Argentina	Daciuk et al. (1981)
	Idaho (USA)	Smith & Belthoff (2001)
	Texas (USA)	Skoruppa et al. (2006)
	Chile	González-Acuña et al. (2006)

	Brazil	Marietto-Gonçalves et al. (2012)
**Siphonaptera**		
Aetheca wagneri	Colorado (USA)	Seery et al. (2003)
	Idaho (USA)	Riding (2010)
Dactylopsylla ignota		Llyuh & Goncharov (2005)
Diamanus montanus		Llyuh & Goncharov (2005)
Echidnophaga gallinacean	California (USA)	Belthoff et al. (2015)
Epitedia inopina		Llyuh & Goncharov (2005)
Epitedia wenmanni	Colorado (USA)	Seery et al. (2003)
Foxella ignota	Colorado (USA)	Seery et al. (2003)
Hoplopsyllus anomalus		Llyuh & Goncharov (2005)
Megabothris clantoni		Llyuh & Goncharov (2005)
Megabothris exilis		Llyuh & Goncharov (2005)
Megabothris wagneri		Llyuh & Goncharov (2005)
Meringis hubbardi	Idaho (USA)	Riding (2010)
Neopsylla hamiltoni	Alberta (Canada)	Brown (1944)
Nosopsyllus fasciatus	California (USA)	Schwan (1984)
Opisocrostis tuberculatus		Llyuh & Goncharov (2005)
Oropsylla hirsuta	Colorado (USA)	Seery et al. (2003)
Oropsylla idahoensis	Alberta (Canada)	Brown(1944)
		Llyuh & Goncharov (2005)
Polygenis (Polygenis) biturus	Tucumán (Argentina)	Lareschi et al., (2016)
Polygenis (Polygenis) platensis	Santa Cruz (Argentina)	Lareschi et al., (2016)
Pulex irritans	Idaho, Oregon, Washington, Colorado (USA)	Belthoff et al. (2015)
Pulex simulans	Idaho, Oregon, Washington, Colorado (USA)	Belthoff et al. (2015)
Rectofrontia fraterna	Alberta, Canada	Brown (1944)
Rhadinopsylla fraterna		Llyuh & Goncharov. (2005)
Thrassis fotus	Colorado (USA)	Seery et al. (2003)
Thrassis pandorae		Llyuh & Goncharov (2005)
**Diptera**		
Icosta americana	Wisconsin (USA)	Mueller et al. (1969)
	Brazil	Graciolli & Barros de Carvalho (2003)
	Argentina	Liébana et al. (2011)
Icosta rufiventris	Brazil	Fontanelli Vaz & Teixeira (2016)
Ornithoica vicina	Brazil	Fontanelli Vaz & Teixeira (2016)
Carnidae fly	SW Idaho (USA)	Smith (1999)

REFERENCES Alfaleh, F.A.; Alyousif, M.S.; Al-Quraishy, S.; Al-Shawa, Y.R. *Eimeria biarmicus* sp.n. (Apicomplexa: Eimeriidae) infecting falcons from the genus *Falco* in Saudi Arabia. *Parasitol. Res.* **2012**, *110*, 1655–1657.

Andery, D. de A.; Ferreira Junior, F.; Araújo, A.V.; Vilela, D. da R.; Marques, M.; Marin, S.Y.; Horta, R.S.; Ortiz, M.C.; Resende, J.S.; Martins, N.S. Health assessment of raptors in triage in Belo Horizonte, MG, Brazil. *Rev. Bras. Cien. Avic.* **2013**, *15*, 247–256.

Atyeo, W.T; Braasch, N.L.; Norman, L. The feather mite genus *Proctophyllodes* (Sarcoptiformes: Proctophyllodidae). *Bull. Univ. Nebr. State Mus.* **1996**, *39*: 1-354.

Belthoff, J.R.; Bernhardt, S.A.; Ball, C.L.; Gregg, M.; Johnson, D.H.; Ketterling, R.; Price, E.; Tinker, J.K. Burrowing owls, *Pulex irritans*, and plague. *Vector-Borne Zoonotic Dis.* **2015**, *15*, 557-564.

Brown, J.H. Sylvatic plague: the recovery of fleas from the burrowing owl and its burrow in a plague area in Alberta. *Entomol. News* **1994**, *55*, 15-18.

Buscher, H.N. *Echinoparyphium speotyto* sp. n. (Trematoda: Echinostomatidae) from the burrowing owl in Oklahoma, with a discussion of the genus *Echinoparyphium*. *J. Parasitol.* **1978**, *64*, 52-58.

Daciuk, J.; Cicchino A.C.; Mauri, R.; Capri, J.J. Notas faunísticas y bioecológicas de Península Valdes y Patagonia. XXIV. Artrópodos ectoparásitos de mamíferos y aves colectados en la Península Valdes y alrededores (Provincia de Chubut, Argentina). *Physis C* **1981**, *39*, 41-48.

da Silva, A.S.; Zanette, R.A.; Lara, V.M.; Gressler, L.T.; Carregaro, A.B.; Santurio, J.M.; Monteiro, S.G. Gastrointestinal parasites of owls (Strigiformes) kept in captivity in the Southern region of Brazil. *Parasitol. Res.* **2009**, *104*, 485–487.

de Almeida Pedroso, L.G.; Hernandes, F.A. New records of feather mites (Acariformes: Astigmata) from non- passerine birds (Aves) in Brazil. Check List. Biodiv. *Data J*. **2016**, *12*, 2000.
Drago, F.B.; Lunaschi, L.I.; Cabrera, N.E.; Barbieri, L. Helminth parasites of four species of strigiform birds from Central and Northeastern Argentina. *Rev. Arg. Parasitol*. **2015**, *4*, 15-23.
Dusek, R.J.; Iko, W.M.; Hofmeister, E.K. Occurrence of West Nile virus infection in raptors at the Salton Sea, California. *J. Wildl. Dis*. **2010**, *46*, 889-895.
Fontanelli-Vaz, F.; Teixeira, V.N. New records of three hippoboscid species on newly captured birds from nature in Paraná, Brazil. Rev. Bras. *Parasitol. Vet*. **2016**, *25*, 501-503.
Franson, J.C. Protozoal hepatitis in a western burrowing owl (*Athene cunicularia hypugaea*). *Southw. Nat*. **2017**, *62*, 75–77.
González-Acuña, D.; Rodrigo Muñoz, C.; Cicchino, A.; Figueroa-R., R.A. Lice of Chilean owls: a first description. *J. Raptor Res*. **2006**, *40*, 301-302.
Goulart, T.M. *Ácaros asociados à "avoante" Zenaida auriculata (Des Murs, 1847) na regiao de Campinas-SP, Brasil*. Tesis de Maestría, Universidade Estadual de Campinas, Brazil, **2011**.
Graciolli, G.; Barros de Carvalho, C.J. Hippoboscidae (Diptera, Hippoboscoidea) no Estado do Paraná, Brasil: chaves de identificação, hospedeiros e distribuição geográfica. *Rev. Bras. Zool*. **2003**, *20*, 667-674.
Hernández-Orts, J.S.; Pinacho-Pinacho, C.D.; García-Varela, M.; Kostadinova, A. *Maritrema corai* n. sp. (Digenea: Microphallidae) from the white ibis *Eudocimus albus* (Linnaeus) (Aves: Threskiornithidae) in Mexico. *Parasitol. Res*. **2015**, *115*, 547–559.
Jellison, W.L. The burrowing owl as a host of the argasid tick, *Ornithodoros parkeri*. *Public Health Rep*. **1940**, *55*, 206-208.
Kinsella, J.M.; Foster, G.W.; Forrester, D.J. Parasitic helminths of five species of owls from Florida, U.S.A. *Comp. Parasitol*. **2001**, *68*, 130-134.
Kurey, W.J. *Ectoparasitic Acarina (Analgoidea) from non-passeriform birds of North America*. M.S. Thesis. Youngstown State University, Youngstown, OH, USA, 1976.
Lareschi, M.; Sánchez, J.; Autino, A. A review of the fleas (Insecta: Siphonaptera) from Argentina. *Zootaxa* **2016**, *4103*, 239–258.
Liébana, M.S.; Santillán, M.Á.; Cicchino, A.C.; Sarasola, J.H.; Martínez, P.; Cabezas, S.; Bó, M.S. Ectoparasites in free-ranging American kestrels in Argentina: implications for the transmission of viral diseases. *J. Raptor Res*. **2011**, *45*, 335–341.
Llyuh, M.P.; Goncharov. A.I. On the fleas IDS. Caucasian *Ornithol. Gaz*. **2005**, *17*, 5-8 [in Bielorussian].
Loomis, R.B. The chigger mites of Kansas (Acarina, Trombiculidae). *Univ. Kansas Sci. Bull*. **1956**, *37*, 1195-1442.
Marietto-Gonçalves, G.A.; Martins, T.F.; Andreatti Filho, R.L. Chewing lice (Insecta, Phthiraptera) parasitizing birds in Botucatu, SP, Brazil. *Rev. Bras. C. Vet*. **2012**, *19*, 206-212.
Mascarenhas, C.S.; Bernardon, F.F.; Gastal, S.; Müller, G. Checklist of the parasitic nasal mites of birds in Brazil. *Syst. App. Acarol*. **2018**, *23*, 1672-1692.
Mueller, N.S.; Mueller, H.C.; Berger, D.D. Host records and phenology of louse-flies on Wisconsin birds. Trans. *Wisconsin Acad. Sci. Arts Lett*. **1969**, *57*,189–207.
Need, J.T.; Dale, W.E.; Keirans, J.E.; Dasch, G.A. Annotated list of ticks (Acari: Ixodidae, Argasidae) reported in Peru: distribution, hosts, and bibliography. *J. Med. Entomol*. **1991**, *28*, 590-597.
Pence, D.B.; Bergan, J.F. Hypopi (Acari: Hypoderatidae) from Owls (Aves: Strigiformes: Strigidae). *J. Med. Entomol*. **1996**, *33*, 828-834.
Philips, J.R. A review and checklist of the parasitic mites (Acarina) of the Falconiformes and Strigiformes. *J. Raptor Res*. **2000**, *34*, 210-231.
Redig, P.T.; Cooper, J.E.; Remple, D.; Hunter, D.B. *Raptor Biomedicine*. Minneapolis, University of Minnesota Press, Minneapolis, USA, **1993**.
Riding, C.S. *Effects of old nest material on occupancy and reuse of artificial burrows, and breeding dispersal by burrowing owls (*Athene cunicularia*) in southwestern Idaho*. PhD Thesis, Boise State University, Boise, USA, **2010**.
Schwan, T.G. *Nosopsyllus fasciatus* parasiting house mice on Southeast Farallon Island, California (Siphonaptera: Ceratophyllidae). *Pan-Pacific Entomol*. **1984**, *60*, 345-349.

Seery, B.D.; Biggins, D.E.; Montenieri, J.A.; Enscore, R.E.; Tandia, D.T.; Gage, K.L. Treatment of black-tailed prairie dog burrows with deltamethrin to control fleas (Insecta: Siphonaptera) and plague. *J. Med. Entomol*. **2003**, *40*, 718-722.
Silva, T.M.; Sakai-Okamoto, A.; Firmino da Silva, L.A.; Domeneghetti-Smaniotto, B.; Da Silva, R.J.; Andreatti-Filho, R.L. New record of *Pelecitus* sp. (Nematoda, Onchocercidae) as a parasite of *Athene cunicularia* (Strigiformes, Strigidae) in southeastern Brazil. Rev. Bras. *Parasitol. Vet*. **2014**, *23*, 274-275.
Skoracki, M.; Unsoeld, M.; Marciniak, N.; Sikora, B. Diversity of quill mites of the family Syringophilidae (Acari: Prostigmata) parasitizing owls (Aves: Strigiformes) with remarks on the host-parasite relationships. *J. Med. Entomol*. **2016**, *53*, 815-826.
Skoruppa, M.K.; Pearce, B.; Woodin, M.C.; Hickman, G.C. Ectoparasites of burrowing owls (*Athene cunicularia hypugaea*) wintering in Southern Texas. *Texas J. Sci*. **2006**, *58*, 73-78.
Smith, B.W. *Nest-site selection, ectoparasites, and mitigation techniques: studies of burrowing owls and artificial burrow systems in southwestern Idaho*. MS Thesis, Boise State University, Boise, USA, **1999**.
Smith, B.W.; Belthoff, J.R. Identification of ectoparasites on burrowing owls in southwestern Idaho. *J. Raptor Res*. **2001**, *35*, 159-161.
Thompson, G.B. XXX.-A list of the type-hosts of the Mallophaga and the lice described from them. *Ann. Mag. Nat. Hist*. **1950**, *3*, 365-382.
Vitaliano, S.N.; Soares, H.S.; Pena, H.F.J.; Dubey, J.P.; Gennari, S.M. Serologic evidence of Toxoplasma gondii infection in wild birds and mammals from southeast Brazil. *J. Zoo Wildl. Med*. **2014**, *45*, 197-199.

Iconography of ectoparasites found in burrowing owls from Bahia Blanca, Argentina.

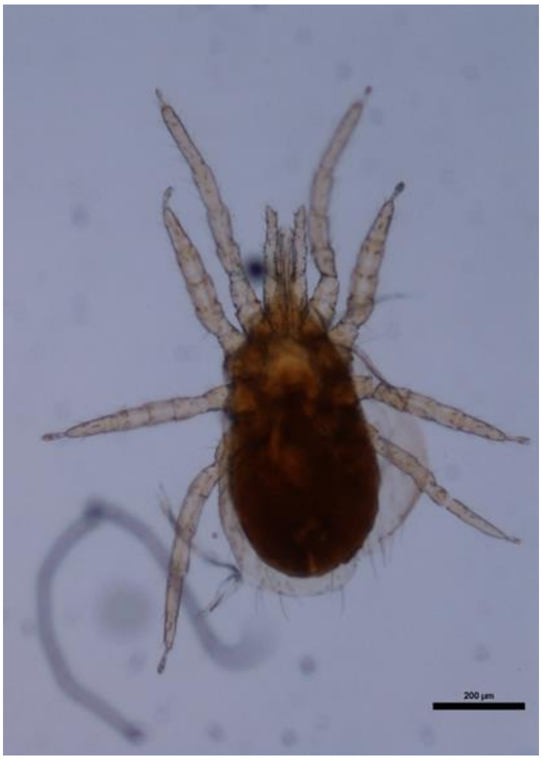

Figure S1. General view of a mite collected from *Athene cunicularia* from Bahia Blanca (Argentina): family Laelapidae (order Mesostigmata), scale bar: 200 µm.

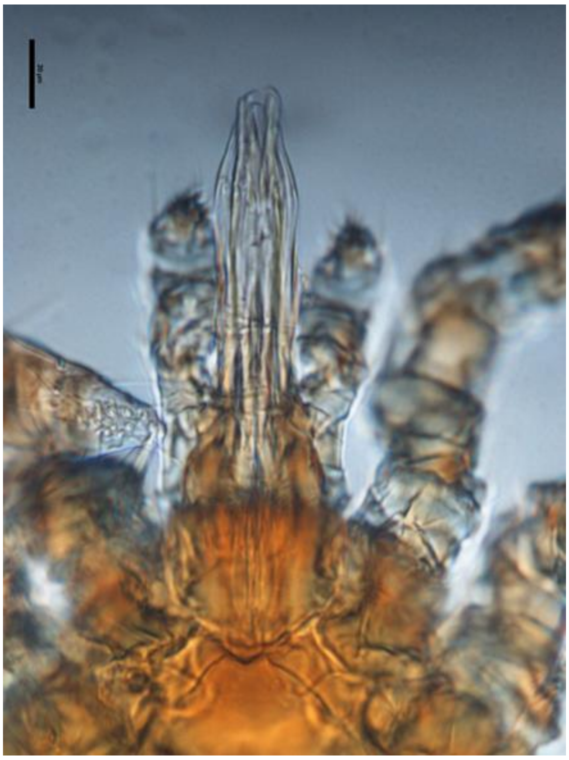

Figure S2. Laelapid mite collected from *Athene cunicularia* from Bahia Blanca (Argentina): detail of the gnatosoma, scale bar: 20 µm.

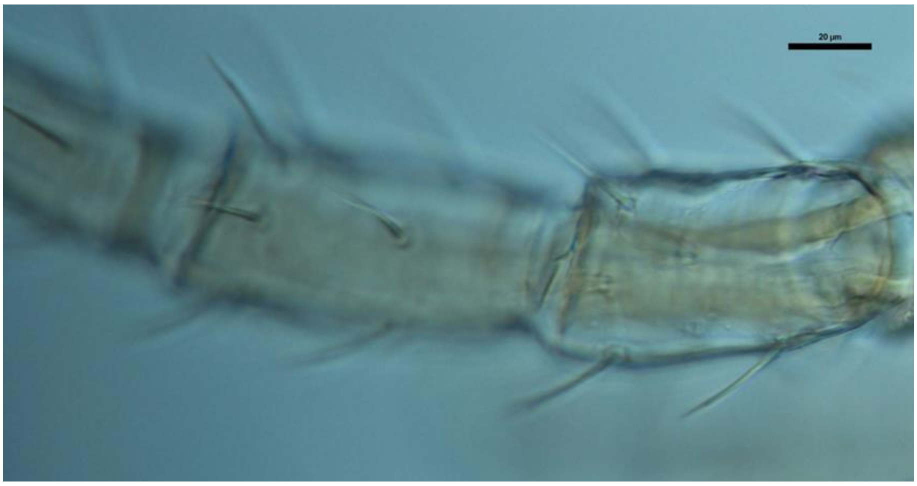

Figure S3. Laelapid mite collected from *Athene cunicularia* from Bahia Blanca (Argentina): ventral setae of tibia I, scale bar: 20 μm.

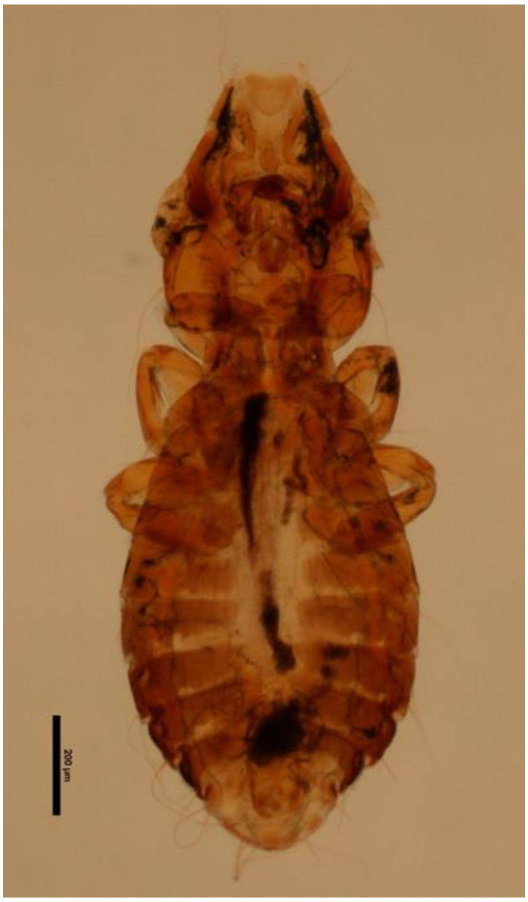

Figure S4. *Strigiphilus speotyti*: habitus of an adult male, scale bar: 200 μm.

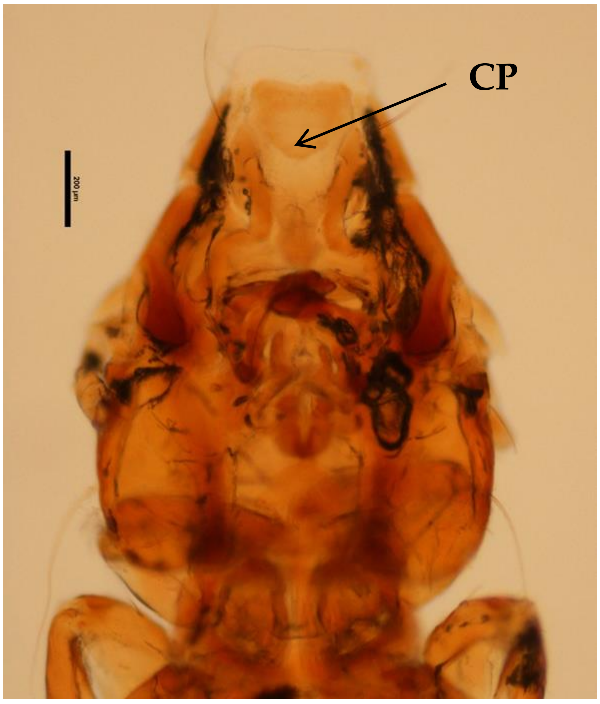

Figure S5. *Strigiphilus speotyti*: detail of the head and clipeal plate (CP), scale bar: 200 µm.

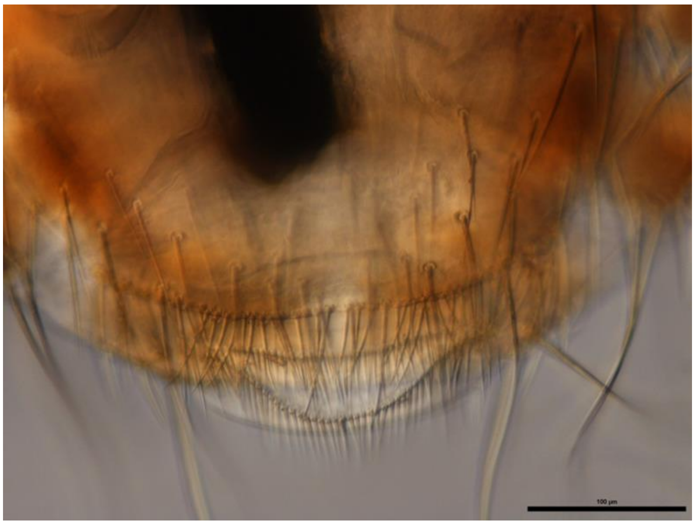

Figure S6. *Colpocephalum pectinatum*. Female genitalia, scale bar: 100 µm. A complete description of *Colpocephalum pectinatum* collected from Bahia Blanca, Argentina can be found in Liébanas et al. *Arthropod Struct Develop*. **2021**, *64*, 101085.

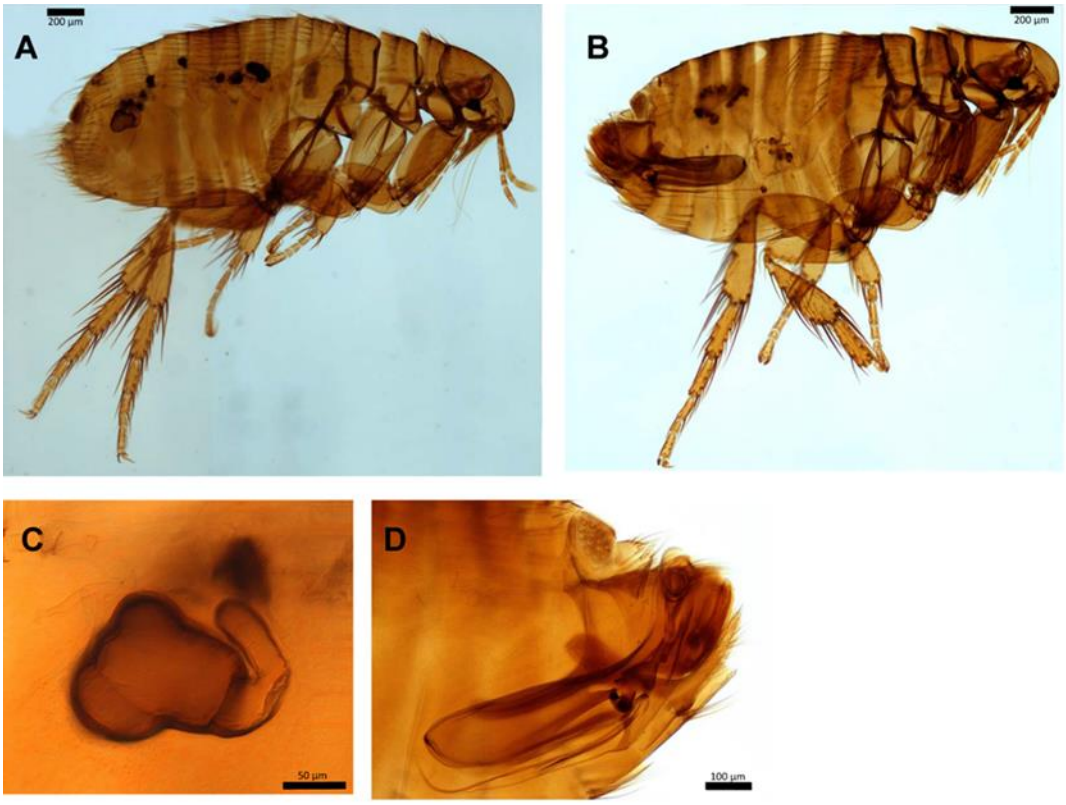

Figure S7. *Polygenis plantensis*. **A**. General view of an adult female. **B**. General view of an adult male. **C**. Spermatheca. **D**. male genitalia. Specimens collected on *Athene cunicularia* from Bahia Blanca, Argentina.
